# Cross-and-Diagonal Networks: An Indirect Self-Attention Mechanism for Image Classification

**DOI:** 10.3390/s24072055

**Published:** 2024-03-23

**Authors:** Jiahang Lyu, Rongxin Zou, Qin Wan, Wang Xi, Qinglin Yang, Sarath Kodagoda, Shifeng Wang

**Affiliations:** 1 School of Optoelectronic Engineering, Changchun University of Science and Technology, Changchun 130022, China; 2023200079@mails.cust.edu.cn (J.L.); 2021000613@mails.cust.edu.cn (R.Z.); 2021001150@mails.cust.edu.cn (Q.W.); 2022000512@mails.cust.edu.cn (W.X.); 2022003119@mails.cust.edu.cn (Q.Y.); 2 Faculty of Engineering & Information Technology, University of Technology Sydney, Sydney, NWS 2007, Australia; sarath.kodagoda@uts.edu.au; 3 Zhongshan Institute of Changchun University of Science and Technology, Zhongshan 528400, China

**Keywords:** image classification, computer vision, self-attention mechanism, CNN

## Abstract

In recent years, computer vision has witnessed remarkable advancements in image classification, specifically in the domains of fully convolutional neural networks (FCNs) and self-attention mechanisms. Nevertheless, both approaches exhibit certain limitations. FCNs tend to prioritize local information, potentially overlooking crucial global contexts, whereas self-attention mechanisms are computationally intensive despite their adaptability. In order to surmount these challenges, this paper proposes cross-and-diagonal networks (CDNet), innovative network architecture that adeptly captures global information in images while preserving local details in a more computationally efficient manner. CDNet achieves this by establishing long-range relationships between pixels within an image, enabling the indirect acquisition of contextual information. This inventive indirect self-attention mechanism significantly enhances the network’s capacity. In CDNet, a new attention mechanism named “cross and diagonal attention” is proposed. This mechanism adopts an indirect approach by integrating two distinct components, cross attention and diagonal attention. By computing attention in different directions, specifically vertical and diagonal, CDNet effectively establishes remote dependencies among pixels, resulting in improved performance in image classification tasks. Experimental results highlight several advantages of CDNet. Firstly, it introduces an indirect self-attention mechanism that can be effortlessly integrated as a module into any convolutional neural network (CNN). Additionally, the computational cost of the self-attention mechanism has been effectively reduced, resulting in improved overall computational efficiency. Lastly, CDNet attains state-of-the-art performance on three benchmark datasets for similar types of image classification networks. In essence, CDNet addresses the constraints of conventional approaches and provides an efficient and effective solution for capturing global context in image classification tasks.

## 1. Introduction

Image classification is widely applied in practical applications such as autonomous driving, medical diagnoses, and security monitoring in the field of computer vision. However, accurate image classification still faces many challenges due to the complexity and variability of images. Over the past few decades, numerous algorithms and methods for image classification have been proposed. One commonly used approach is to employ traditional machine learning algorithms, which often rely on handcrafted feature extractors utilizing low-level features such as color histograms, texture features, and shape descriptors. However, these methods require domain expertise and significant manual effort in selecting and extracting appropriate features, limiting their performance on complex scenes and large-scale datasets. In contrast, the rapid advancements in deep learning techniques have brought significant breakthroughs in image classification. Deep learning models, particularly convolutional neural networks (CNNs), have the capability to extract high-level abstract features from raw pixels and effectively classify images. Furthermore, an image classification task is the fundamental prerequisite for various downstream tasks, such as object detection, image segmentation, and so on. Thus, many deep learning-based image classification models have been proposed and applied to various fields, including wearable robots, geological exploration, medical diagnoses, and crop detection [1,2,3,4]. With the continuous development of sensors, the image quality of various types has been constantly improving. In recent years, many hyperspectral image classification models have been proposed [5,6,7,8]. Compared to RGB images, hyperspectral imagery can provide more accurate and detailed land object classification results by fully exploiting spectral information, thereby offering support for applications in various domains. In addition, many impressive models have been proposed in fields such as infrared imagery [9,10] and medical imaging [11,12], making significant contributions to their respective areas of application. Meanwhile, several non-deep learning-based approaches to image classification have emerged [13,14,15]. The mutual promotion between these two approaches actively contributes to image classification techniques in tandem.

**Figure 1:**
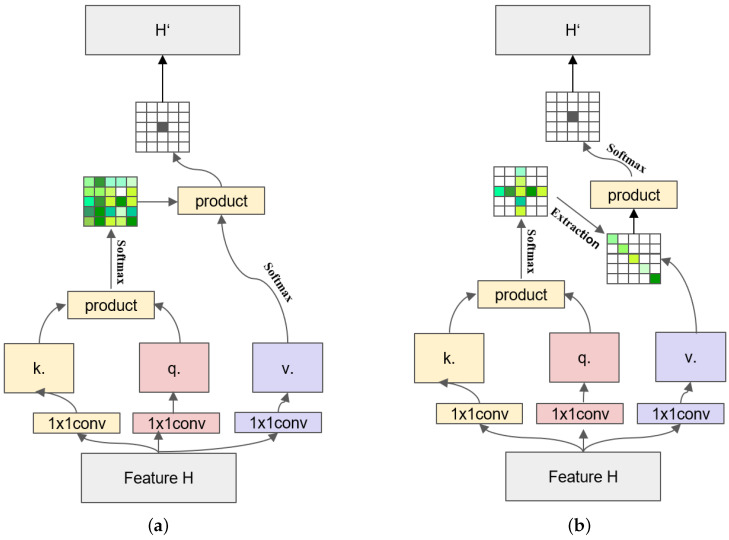
Comparison of non-local and cross-diagonal block. (**a**) The details of non-local block module. (**b**) The details of cross and diagonal block module.

Fully convolutional neural (FCN) networks have achieved remarkable success in recent years. However, the inherent limitations of FCNs, such as restricted receptive fields and inadequate contextual information, have impeded their progress and constrained further advancements. In addition, conventional self-attention mechanisms typically introduce direct dependencies between a pixel and all other pixels in an image, leading to increased computational complexity and potentially limiting inference speed. These challenges are significant barriers to the further development and practical application of fully convolutional (FCN) networks and self-attention methods. Through extensive experiments, as shown in Figure 1a,b, we have found that the above problem can be effectively alleviated by modifying the computation process of the non-local block [16] in self-attention from a direct to an indirect method. Specifically, as shown in Figure 1b, the proposed indirect self-attention block can split one computation in the original non-local block into two computations through two successive operations from two different directions (cross and diagonal) to establish the long-distance dependence of a single pixel point on the rest of the pixels. In this way, pixel-level contextual information can be summarized from the remaining points in the image. Modifying the computation method can significantly decrease the computational complexity of the self-attention operation. The original non-local block generates a densely weighted attention map of size H×W. In contrast, the indirect self-attention network generates a weighted H+W−1 of the attention graph. Therefore, compared to the non-local block, our indirect self-attention reduces the computational complexity from O(H×W)×(H×W) to $O_{2}\left(H+W-1\right)\times \left(H+W-1\right)$. In summary, CDNet has several advantages:

- It can aggregate contextual information over long distances so that the entire network has rich local feature information while taking global features into account, improving network performance.
- In contrast to the non-local block, CDNet significantly simplifies the computational complexity of the network, resulting in a more streamlined architecture. This simplification enhances the GPU friendliness of the network, thereby improving the overall utilization efficiency.
- The cross and diagonal block as a plug-and-play module can be seamlessly integrated into the framework of fully convolutional neural networks. This integration is straightforward, requiring minimal modification to the existing network architecture.

## 2. Related Works

Recently, there has been a growing emphasis on image classification networks that amalgamate attention mechanisms and convolution, alongside the conventional networks mentioned earlier. It captures the interrelationships among channels via two processes: squeezing and exciting. It recalibrates the strength of feature responses between channels by using the network’s global loss function. SK-Net [17] draws inspiration from the Inception block and SE block while incorporating multi-scale feature representations. It presents various convolutional kernel branches to acquire feature map attention across multiple scales, enabling the network to concentrate more on significant scale-specific features. In 2020, ECA-Net was proposed by Qilong Wang et al. [18]. The authors found that the computational complexity of channel attention can be lowered by avoiding dimension reduction, all while achieving high accuracy. They presented a self-adaptive and selective convolutional operation to accomplish this. Similarly, in the same year, Hang Zhang et al. introduced Split-Attention Networks [19]. In 2017, Vaswani and colleagues introduced the Transformer [20]. It demonstrated outstanding performance in natural language processing. Although natural language processing (NLP) and image classification are relatively independent fields, the self-attention mechanism has played a crucial role in various tasks. Subsequently, several variants have been proposed, including the vision Transformer (ViT) [21], which achieved state-of-the-art results on the ImageNet dataset [22]. ViT, proposed by Google, is a model that applies the Transformer to image classification. Although it was not the first paper to apply the Transformer to visual tasks, it has become a milestone work for the application of the Transformer in computer vision due to its “simplicity”, good performance, and strong scalability (larger models achieve better results). ViT has demonstrated excellent performance in various computer vision tasks, including object detection and semantic segmentation. And the Swin Transformer [23] improved upon the ViT. The key distinctions between the Swin Transformer and ViT lie in their model architecture and processing strategies. The Swin Transformer leverages a novel window-based mechanism and block processing.

In addition, numerous variants of transformer models based on the self-attention mechanism have been proposed and applied in various other domains. In 2022, X. Chen et al. proposed the Class-Guided Swin Transformer [24] based on the Swin Transformer and applied it to the semantic segmentation of remote sensing images. Variants based on the Swin Transformer also play significant roles in different domains. For instance, DS-TransUNet [25] and SQ-Swin [26] are notable examples. The former has been applied in the field of medical image segmentation, while the latter has been used in the context of food-safety-related issues. In the same year, Cross-Stream Attention [27] was proposed, which leverages optical flow from infrared data to address motion recognition in low-light conditions. The self-attention mechanism in Transformers has also become a hot topic in recent years. In 2023, J. Chen, S. Yu, and J. Liang proposed Cross-layer Self-attention [28], which is utilized to address the problem of fine-grained image classification. Subsequently, SelfAT-Fold [29] was proposed for protein folding recognition. There are many other related networks [30,31,32,33]. Meanwhile, there is an increasing trend of integrating cross-modal thinking with attention mechanisms in high-spectral image processing tasks [34,35,36]. Liu and Peng et al. proposed RPCL-FSL, which incorporates supervised contrastive learning (CL) and FSL into an end-to-end network to perform small-sample HSI classification. And it imposes triple constraints on prototypes of the support set, i.e., CL-, self-calibration (SC)-, and cross-calibration (CC)-based constraints. Similarly, Xi et al. have also applied cross-modal thinking to high-spectral image processing tasks by proposing a Cross-scale Graph Prototype Network (X-GPN) to achieve semi-supervised high-quality high-spectral image classification tasks. In the same year, Zhao and Qin et al. proposed a hyperspectral classification framework based on a multi-attention Transformer (MAT) and adaptive superpixel segmentation-based active learning (MAT-ASSAL). It also solves the problem of CNN sensory field limitation by a multi-attention Transformer. Their emergence undoubtedly signifies that a Transformer and self-attention mechanisms have become research hotspots in computer vision and other fields. The aforementioned methodologies have demonstrated remarkable achievements in image classification as well as its associated tasks, substantially enhancing the precision of pertinent datasets when juxtaposed with convolutional neural networks (CNNs).

## 3. Methods

**Figure 2:**
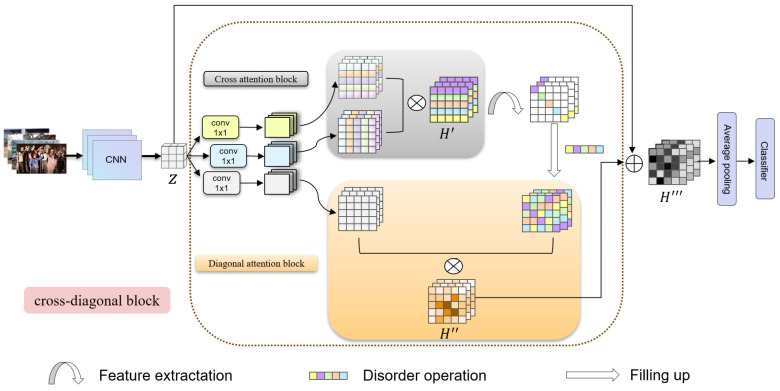
Overview of the proposed CDNet.

In this section, we will examine the particular aspects of indirect self-attention. The process can be broadly divided into two phases. Remote dependencies between positions have been effectively established using these two computations, thereby obtaining abundant global context information. The implementation of CDNet is proposed to address the issue of insufficient global information in convolutional computation. As illustrated in Figure 2, typical attention mechanisms compute the weights of feature information at the current position directly. Our approach aims to expand the coverage of feature information contained in a single position by connecting the operations of row–column and diagonal computation through concatenation, thus solving the problem of overly focusing on the local information brought by the full convolutional operation.

### 3.1. Overall Approach

As shown in Figure 2, the image data are fed into the input of the convolutional neural network, and after multilayer processing, a high-dimensional feature of size L×W, denoted as *Z*, is obtained, and *Z* is used as the input to the dc block. To keep the algorithm efficient, it runs through three sets of 1×1 convolutions with the separate dimensionality reduction in *Z*. After downsizing, we obtain three sets of feature maps with the same size and 1/4 of the original number of channels, denoted as k,q,v. The standard self-attention process involves calculating the dot product of *k* and *q* to obtain the feature map for a long-distance dependency and self-relationship. This is combined with *v* to achieve coherent global contextual information aggregation within the cross and diagonal block. Such aggregation is obtained via both cross and diagonal attention blocks in tandem with the diagonal operation. The feature map produced by implementing the cross attention block is designated as $H^{\prime }$. It combines data from the corresponding row and column for every pixel on the map. Subsequently, the feature map $H^{\prime }$ is fed into the diagonal attention block, resulting in a new feature mapping H″. Therefore, each pixel in $H^{\prime \prime }$ aggregates pixel information from different rows and columns, incorporating all the information from the respective row and column. This process indirectly achieves the aggregation of global context information and creates a wide range of remote dependencies. The local feature and the global context are concatenated as the output feature of the whole network, denoted as $H^{\prime \prime \prime }$. Finally, the feature is passed through the classifier after performing average pooling to obtain the output result.

The Affinity operation involves obtaining separate row and column vectors from the input feature maps, followed by vector multiplication between the two vectors:

(1) $$\begin{array}{c} Af\;f\;inity=\sum_{i=0}^{n}\left(a_{i}*b_{i}\right) \end{array}$$

The vectors a and b correspond to row and column vectors within the feature map. The parameter n represents the total number of vectors. The extraction procedure is implemented to retrieve the elements of the feature map that lie on the diagonal.

(2) $$\begin{array}{c} D=diag\left(a_{11},\ldots ,a_{nn}\right)=\sum_{i=1}^{n}P_{\left(i\right)}AP_{\left(i\right)} \end{array}$$

where $P_{\left(i\right)}$ is the projection on the i-th coordinate:

(3) $$\begin{array}{c} P_{(i)jk}=\delta_{j}\delta_{jk}\;\left(i,j,k\in \left\{1,\ldots ,n\right\}\right) \end{array}$$

and δ is the Kronecker delta (1 for the same index values, otherwise 0).

PDO refers to the process of padding in disordered order. It involves duplicating the extracted elements as L×W and padding them in a disordered order to generate a new feature map with L×W dimensions. This process is then repeated C times in order to obtain a feature map with the same dimensions as before extraction, where C is the number of channels.

### 3.2. Cross Attention

**Figure 3:**
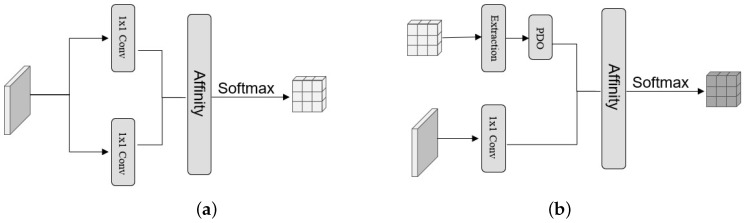
Overviews of cross attention and diagonal attention. (**a**) The details of cross attention block. (**b**) The details of diagonal attention block.

As illustrated in Figure 3a, the proposed cross attention model aims to create a feature map that consolidates feature information from all pixels in the same row–column as the pixel in question. This process equips each pixel with contextual information related to its row–column position. Specifically, the initial step involves feeding the feature map *H* (with dimensions L×W×C) into two 1×1 convolutional layers, resulting in two outputs, namely *M* (with dimensions L×W×C/4) and *N* (with dimensions L×W×C/4), respectively. Then, we can gather the feature information of pixels that travel with it from any pixel in *M* using the Affinity operation. We retrieve the row vector $M_{i}$ and its corresponding column vector from *N*, denoted as $N_{i}$. Then, we combine $M_{i}$ with $N_{i}$, and perform vector multiplication as follows:

(4) $$\begin{array}{c} Q=M_{i}*N_{i}^{T} \end{array}$$

after taking the transpose of $N_{i}$ to obtain $N_{i}^{T}$ and projecting $N_{i}$ onto $M_{i}$ to obtain *Q*, which represents the correlation between the two vectors, we apply a softmax operation on *Q* in that dimension to generate new feature mapping.

### 3.3. Diagonal Attention

A novel attention map is generated after the cross attention block. Each element, $a_{ij}$, possesses a varying degree of correlation with the other pixels within the $j_{th}$ column of the $i_{th}$ row. This grants $a_{ij}$’s attention range the capability to encompass all other elements on the same row or column. Then, we suggest using a diagonal attention module built on the cross attention module, presented in Figure 3b, to establish a comprehensive long-distance dependency and acquire more extensive global context information. The diagonal attention block comprises two primary paths. The first path, known as the k&q path, executes an extraction operation on the attention map obtained by the cross attention block. This operation extracts the elements situated on its diagonal line. Subsequently, the PDO operation is employed for padding the diagonal elements obtained, generating a new feature map of the same size as the original. The second path is the v-path, where the feature map obtained after the full convolutional network is utilized again and it is fed into the convolutional layer of the 1×1 filter, and then the column vectors in the obtained feature map are vectorially multiplied by the row vectors in the feature map obtained in the k&q path as follows:

(5) $$\begin{array}{c} P=S_{j}*T_{J} \end{array}$$

where $S_{j}$ originates from the *k* and *q* paths, $T_{j}$ originates from the *v* path, and *P* represents the intended attention graph that includes global information.

Overall, our approach compensates for the previous deficiency of global information in complete convolutional neural networks. It indirectly broadens the attention range of the network by implementing two attention modules, thus establishing a mechanism of attention with a wider scope at a greater distance. Meanwhile, when comparing it to the non-local one, the original computational complexity of $O{\left(H\times W\right)}^{2}$ is reduced to $O_{2}{\left(H+W-1\right)}^{2}$.

## 4. Experiment

Three widely accepted datasets, including Cifar10, Cifar100, and ImageNet, are utilized in our image classification experiments to evaluate the efficiency and effectiveness of our network. Experiments demonstrate that CDNet can attain the state-of-the-art level among comparable attention networks and even surpass some substantial models in tasks related to image classification. Additionally, it reduces computational complexity compared to previous networks utilizing the self-attention mechanism.

### 4.1. Details of the Experiments

- **CIFAR-10**: CIFAR-10 is a dataset of color images that represents a broader range of universal objects. It is a limited dataset designed for identifying general objects, arranged by Alex Krizhevsky and Ilya Sutskever. It includes 10 categories of RGB color images. The dataset contains 50,000 training images and 10,000 test images, with each category consisting of 6000 images measuring 32 × 32 pixels.
- **CIFAR-100**: The CIFAR100 dataset comprises 100 classes, each containing 600 color images of dimensions 32 × 32. Among these images, 500 serve as training data while the remaining 100 serve as test data, resulting in 60,000 images. Each image is assigned two labels: fine labels and coarse labels. These labels indicate the detailed and general classification of the image, respectively.
- **Fashion-MNIST**: Fashion-MNIST is a dataset comprising 28 × 28 grayscale images of 70,000 fashion products from 10 categories, with 7000 images per category. The training set has 60,000 images, and the test set has 10,000 images. Fashion-MNIST shares the same image size, data format, and structure of training and testing splits with the original MNIST.
- **ImageNet**: We employed the ImageNet1K dataset, comprising 1.28 million images for training and 50 K for validation across 1000 classes.

Standard like-for-like data enhancements were employed in the experiments. All of the experiments were carried out on the four datasets. The label-smoothing regularization was employed during the training process. The SGD strategy was utilized during parameter optimization with a momentum value of 0.9, an initial learning rate of 0.1, and a weight decay value of 5 × 10^−4^. It should be noted that when training on the ImageNet1k dataset, the values of the initial learning rate and weight decay were adjusted to 0.2 and 1 × 10^−4^, respectively. Regarding the training strategy, we conducted training on the CIFAR dataset for 400 epochs, with the learning rate decreasing by a factor of 10 every 60 epochs. For the ImageNet1k dataset, we followed the same strategy as described in reference [37], training for 100 epochs and a single 224 × 224 crop for evaluation, except R-Mix [38] and ResMLP-36 [39]. All the networks were trained on a single NVIDIA GTX A6000 GPU. The experimental results represent the average value obtained from three independent trials.

### 4.2. Evaluating Indicator

In this paper, in addition to accuracy as a common evaluation metric, several other metrics are often employed to evaluate the performance of a classifier, including precision, recall, and specificity.

**Figure 4:**
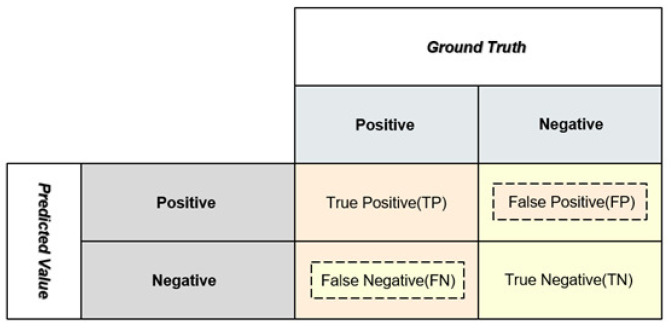
Distinguishing attributes of classification results based on predictions and ground truth.

As shown in Figure 4, based on the prediction value and ground truth, the classification results are assigned four attributes: true positive (TP), False Positive (FP), False Negative (FN), and true negative (TN).

(6) $$\begin{array}{c} Precision=\frac{TP}{TP+FP} \end{array}$$

precision is defined as the proportion of samples predicted as positive that belong to the positive class. It is based on the prediction results and measures the correctness of positive predictions. It focuses on the accuracy of positive prediction results.

(7) $$\begin{array}{c} Recall=\frac{TP}{TP+FN} \end{array}$$

recall, in contrast, is a metric that describes the proportion of positive samples correctly identified among all actual positive samples. It is based on the true samples and measures the proportion of correctly predicted positive samples among the true positive samples. It focuses on the completeness of predicting true positive samples.

(8) $$\begin{array}{c} Specificity=\frac{TN}{TN+FP} \end{array}$$

specificity refers to the proportion of predicted negative samples to true negative samples. This indicator is used to distinguish the true negative samples from all predicted negative samples based on true samples.

### 4.3. Cifar Classification

ResNext is a deep learning network employed for image classification, and in this experiment, it is employed as the CNN in CDNet, as shown in Figure 2. Therefore, for both CDNet and ResNext, 18 and 29 denote the convolutional layers’ depth. The experiments were conducted on the CIFAR-10 and CIFAR-100 datasets utilizing distinct networks. The experimental results in Table 1 indicate that the ResNet series and the attention-enhanced networks in this paper exhibit superior performance over other networks. Notably, our proposed method demonstrates a reduced parameter count compared to other attention-based networks. On the CIFAR-10 dataset, the performance of CDNet18 in terms of classification accuracy surpassed that of ResNet18, showcasing a significant enhancement of 0.63%. Notably, the performance of CDNet even outshined that of ResNext29 (16x32d), thereby substantiating its noteworthy efficacy. In regard to the CIFAR-100 dataset, CDNet demonstrated superior accuracy relative to other networks, resulting in a remarkable advancement of 1.67 percentage points over the prior state-of-the-art results. In order to compare the classification results of our network and the baseline more intuitively, we used the weights of both networks to perform inference on the test set and obtained their confusion matrices based on the inference results. The weights from both networks were utilized for conducting inference on the test set, enabling a visual assessment of the classification outcomes between our network and the baseline. The corresponding confusion matrices were derived and are presented below for reference.

**Figure 5:**
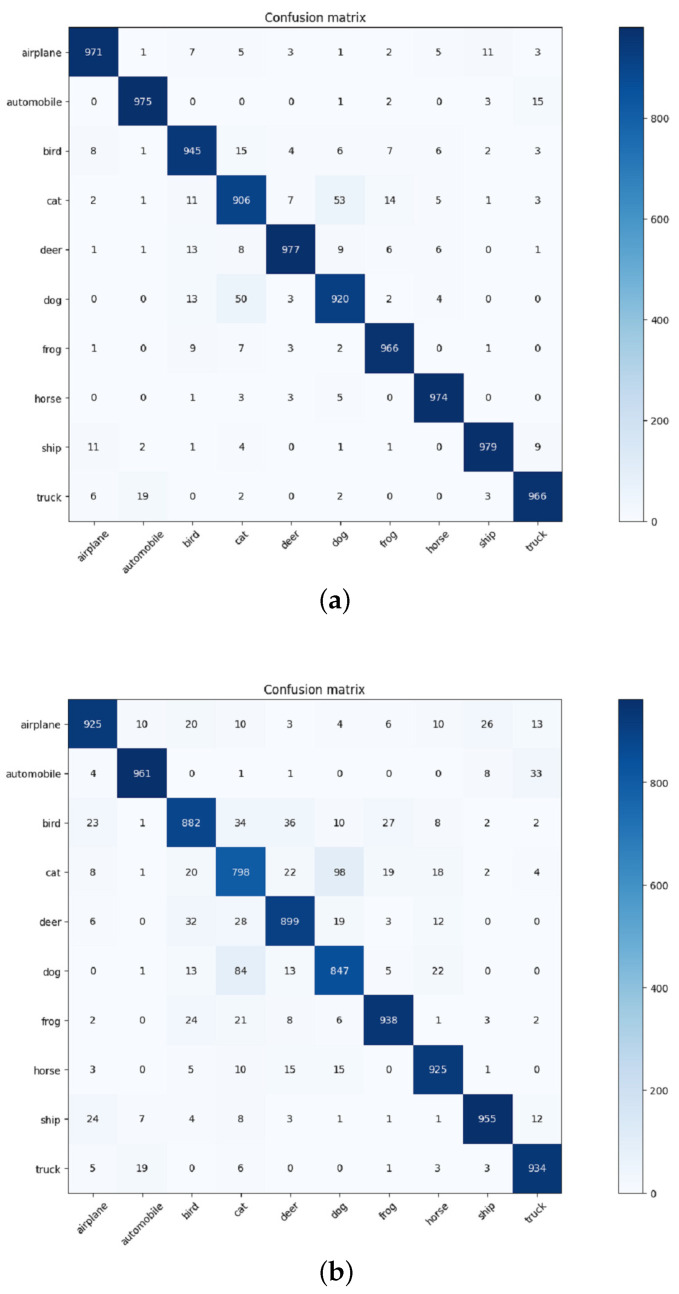
The confusion matrix of ResNext29 and CDNet29 on Cifar-10. (**a**) The confusion matrix of CDNet29 on Cifar-10. (**b**) The confusion matrix of ResNext29 on Cifar-10.

Figure 5a,b show that the horizontal axis denotes the true labels, while the vertical axis represents the predicted results. A higher concentration of values along the diagonal line within the graph indicates more favorable classification outcomes. Table 2 shows the number of accurately classified images for each category in ResNext29 and CDNet29 on the CIFAR-10 dataset. In addition, these data correspond to the data plotted on the diagonal line in Figure 5a,b. Consequently, the classification performance of CDNet outperforms that of the baseline model. Additionally, the precision, recall, and specificity measures for each category in both models could be readily derived by analyzing the confusion matrix. This is shown in Table 3:

Based on the data presented in Table 3, it appears that CDNet outperforms the baseline in terms of precision, recall, and specificity across all categories in the Cifar-10 dataset. These improvements are quite notable and suggest that CDNet may be a promising approach for improving classification performance on this dataset.

### 4.4. Ablation Experiments

Ablation experiments were performed to thoroughly evaluate the impact of individual components within CDNet on classification results, providing detailed insights into their contributions. The first experiment examines the effect of cross and diagonal attention on the CDNet. The purpose of the second experiment is to explore the effect of the convolutional kernel size in the network on the accuracy of the network. Two different baselines were applied on different datasets, ResNext-29 for CIFAR-100 and ResNext-101 for ImageNet1k.

In Table 4 and Table 5, “+C” means that only cross attention is employed, while “+D” means that only cross attention is employed. And “+CD” means that both of them are employed. GFLOPSs stands for Giga Floating-point Operations Per Second, which represents the number of floating-point operations that can be performed in one second at a rate of one billion operations per second. A Top5 error refers to considering the top 5 classes with the highest probabilities in the classification results. If any of the top 5 predicted classes matches the ground truth class, it is considered a correct prediction; otherwise, it is considered a prediction failure. The top-5 error rate is calculated by dividing the number of prediction failures by the total number of samples. On the other hand, a Top1 error denotes considering only the class with the highest probability, while the other conditions remain the same.

By analyzing the experimental results, it was observed that for CDNet, the contribution of cross attention was more significant than that of diagonal attention. It is suggested that this observation may be due to the fact that diagonal attention is positioned as the second step in the cross and diagonal block. It is designed to enhance the contextual information obtained after cross attention. Individually, diagonal attention may not provide highly effective contextual information for the entire network. This confirms the effectiveness of the concept of “indirect” in our indirect self-attention.

Kernel sizes represent the size of the convolutional kernel. Top1 error and Top5 error are defined in the same manner, as shown in Table 4. In addition, it is also speculated that the size of the convolutional kernel in the convolutional layer may affect the experimental results. To examine the impact of different convolutional kernel sizes on the attention mechanism’s effectiveness, the convolution kernel size was systematically varied during the classification experiments. Both cross and diagonal attention mechanisms were applied to conduct classification experiments on CIFAR-100 with various convolutional kernel sizes. Table 6 and Table 7 illustrates the impact of different convolutional kernel sizes on the accuracy of CDNet’s attention mechanism. The results indicate that the highest accuracy is achieved when using a 1×1 kernel size, surpassing the accuracy obtained with a 7×7 kernel size by 0.17%. Based on this finding, the decision was made to utilize 1×1 kernel sizes for all convolutions in CDNet. This adjustment was made to optimize the network’s performance and improve the model’s overall accuracy. Table 3 demonstrates that altering the size of the convolutional kernel in CDNet’s attention mechanism has an impact on the accuracy of the experiments. Specifically, the results indicate that the highest accuracy was achieved when utilizing a kernel size of 1×1. This finding suggests that a 1×1 kernel size is optimal for the attention mechanism in CDNet. It is worth noting that using other kernel sizes in this context led to inferior accuracy results compared to the 1×1 kernel size. This highlights the importance of choosing the appropriate kernel size to perform related tasks best.

### 4.5. Fashion-MNIST Classification

In order to further substantiate the superior performance of CDNet on different datasets, we conducted image classification experiments on the Fashion-MNIST dataset.

In Table 8, Models represents different models, and Top-1 Errors is the same parameter as in Table 9. GFlOPs represents the amount of computation in the model, and Parameters represents the number of parameters in the model.

This experiment serves as a complementary study to the cifar classification experiment, providing a more comprehensive demonstration of the outstanding performance of CDNet on small-scale datasets. Compared to SSGD, CDNet exhibits a 0.08% improvement in accuracy, with lower GFLOPs and parameters.

### 4.6. ImageNet 1k Classification

In order to verify the effectiveness of our network on a larger dataset, image classification experiments were conducted on the ImageNet1k dataset. The experiments demonstrate that our method achieves excellent results on the ImageNet1k dataset.

The experiments show that CDNet is smaller in terms of the number of operations and parameters than the previous model. Their values are slightly higher compared to the baseline network. However, compared with the baseline, CDNet-50 and CDNet-101 improve the accuracy by 1.56% and 0.98%, respectively. The outcome demonstrates superior performance over other variant networks, enhancing the highest accuracy by 0.06%. While our algorithm may exhibit slightly lower accuracy than ResNeXt-101 (64×4), it is essential to consider the significant disparity in model parameters and computational requirements between the two approaches.

### 4.7. Efficiency Experiments

To further validate the efficiency of CDNet, we conducted experiments to calculate its training and inference speeds, and compared them with other algorithms. The experimental results are presented below:

ResNet50 and ResNet101 were employed as baselines in the efficiency experiment. From Table 10, it can be observed that CDNet outperforms other methods in terms of training and inference speed.

## 5. Conclusions

This paper presents CDNet as an indirect self-attention mechanism that can be tessellated into a fully convolutional neural network. The objective is to expand the attention scope of feature maps and establish long-distance dependencies, enhancing the classification accuracy while reducing the computational complexity and parameter count. To validate our proposition, image classification experiments were performed on CIFAR-10, CIFAR-100, and ImageNet1k datasets. It was found that CDNet can achieve an accuracy improvement of 1.16%, 0.66%, and 0.06% over baseline networks on the respective datasets.

## 6. Discussion

Our work has achieved the state of the art in networks with similar structures. However, compared to the mainstream large model approaches today, its performance still lags behind considerably. This also inspires us to work in the future. We can focus on real-time performance and attempt to apply this paper’s “indirect” concept to the large models. In addition, other downstream tasks such as segmentation, detection, and pose estimation can be explored as extensions of our work. 

## Figures and Tables

**Table 1:** Top-1 errors (%, average of 10 runs) on CIFAR. SENet-29, SKNet, and CDNet-29 are all based on ResNeXt-29, 16 × 32 d.

Models	CIFAR-10	CIFAR-100	Parameters
ResNext18	4.45	23.67	11.7M
R-Mix (PreActResNet-18) [38]	3.73	/	/
Resnext29, 16 × 32 d	3.87	18.56	25.2M
Resnext29, 8 × 64 d	3.65	17.77	34.4M
Resnext29, 16 × 64 d	3.58	17.31	68.1M
SENet29 [40]	3.68	17.78	35.0M
SKNet29 [17]	3.47	17.33	27.7M
R-Mix (WideResNet 28-10) [38]	/	15.00	/
SparseSwin [41]	3.57	14.65	17.6M
Ghost-ResNet-56 [42]	7.30	/	**0.4M**
Ghost-VGG-16 [42]	6.30	/	7.7M
WRN28-10 [43]	4.17	19.25	/
Transformer local-attention (NesT-B) [44]	2.80	17.44	68.0M
CDNet18	3.82	22.31	13.3M
CDNet29	**2.31**	**14.32**	27.3M

**Table 2:** The number of accurately predicted images for each class of ResNext29 and CDNet29 on CIFAR-10 dataset.

Labels	ResNext29	CDNet29
Airplane	925	971
Automobile	961	975
Bird	882	945
Cat	798	906
Deer	899	977
Dog	847	920
Frog	938	966
Horse	925	974
Ship	955	979
Truck	934	966

**Table 3:** The precision, recall, and specificity of ResNext29 and CDNet29 on Cifar-10.

Methods	Labels	Precision	Recall	Specificity
	Airplane	0.901	0.925	0.989
	Automobile	0.953	0.961	0.995
	Bird	0.860	0.882	0.984
	Cat	0.806	0.798	0.979
ResNext29	Deer	0.900	0.899	0.989
	Dog	0.860	0.847	0.985
	Frog	0.933	0.938	0.993
	Horse	0.951	0.925	0.995
	Ship	0.942	0.955	0.993
	Truck	0.962	0.934	0.996
	Airplane	0.962	0.971	0.996
	Automobile	0.979	0.975	0.998
	Bird	0.948	0.945	0.994
	Cat	0.903	0.906	0.989
	Deer	0.952	0.977	0.995
CDNet29	Dog	0.927	0.920	0.992
	Frog	0.977	0.966	0.997
	Horse	0.988	0.974	0.999
	Ship	0.971	0.979	0.997
	Truck	0.968	0.966	0.996

**Table 4:** Performance on CIFAR-100 dataset for different attention.

Contributions	GFLOPs	Parameters	Top-1 Errors (%)	Top-5 Errors (%)
Baseline	**4.45 **	**25.2M**	18.56	4.19
+C	4.47	26.4M	16.97	3.93
+D	4.48	25.9M	18.74	4.21
+CD	4.52	27.3M	**16.66**	**3.53**

**Table 5:** Performance on ImageNet 1k dataset for different attention.

Contributions	GFLOPs	Parameters	Top-1 Errors (%)	Top-5 Errors (%)
Baseline	**7.99**	**44.3M**	21.11	8.92
+C	8.00	46.8M	20.57	7.98
+D	8.00	45.6M	21.18	8.23
+CD	8.00	47.5M	**20.13**	**7.34**

**Table 6:** Performance in cross-diagonal block when applying convolution with different kernel sizes on the CIFAR-100 dataset.

Kernal Sizes	Top-1 Errors (%)	Top-5 Errors (%)
1×1	16.66	**3.53**
3×3	**16.63**	3.69
5×5	16.77	3.83
7×7	16.72	3.89

**Table 7:** Performance in cross-diagonal block when applying convolution with different kernel sizes on the ImageNet-1k dataset.

Kernal Sizes	Top-1 Errors (%)	Top-5 Errors (%)
1×1	**20.13**	**7.34**
3×3	20.40	7.74
5×5	20.72	8.06
7×7	20.72	8.16

**Table 8:** Top-1 errors on Fashion-MNIST for different methods.

Models	Top-1 Errors (%)	GFLOPs	Parameters
ResNeXt-50+BAM [45]	17.40	4.31	**25.4M**
ResNeXt-50+CBAM [46]	17.08	4.25	27.7M
SENet-50 [40]	17.02	**4.25**	27.7M
SKNet-50 [17]	16.73	4.47	27.5M
ResNeXt-101+BAM [45]	15.35	8.05	44.6M
ResNeXt-101+CBAM [46]	14.80	8.00	49.2M
SENet-101 [40]	14.62	8.00	49.2M
SKNet-101 [17]	14.20	8.46	48.9M
SSGD(MLP) [47]	17.30	/	30.5M
SSGD(CNN) [47]	13.30	/	51.4M
CDNet-50	16.44	**4.25**	27.3M
CDNet-101	**13.21**	8.00	47.5M

**Table 9:** Top-1 errors on ImageNet1k for different methods.

Models	Top-1 Errors (%)	GFLOPs	Parameters
R-Mix(ResNet-50) [38]	22.61	/	/
ResNeXt-50	22.23	**4.24**	**25.0M**
AttentionNeXt-56 [48]	21.76	6.32	31.9M
ECA-Net [18]	21.08	10.80	57.4M
ResNeXt-50+BAM [45]	21.70	4.31	25.4M
ResNeXt-50+CBAM [46]	21.40	4.25	27.7M
SENet-50 [40]	21.12	4.25	27.7M
SKNet-50 [17]	20.79	4.47	27.5M
ResNeXt-101	21.11	7.99	44.3M
DPN-92 [49]	20.70	6.50	37.7M
DPN-98 [49]	20.20	11.70	61.6M
ResNeXt-101+BAM [45]	20.67	8.05	44.6M
ResNeXt-101+CBAM [46]	20.60	8.00	49.2M
ResMLP-36 [39]	20.30	/	45.0M
SENet-101 [40]	20.58	8.00	49.2M
SKNet-101 [17]	20.19	8.46	48.9M
CDNet-50	20.66	4.25	27.3M
CDNet-101	**20.13**	8.00	47.5M

**Table 10:** Training or inference speed (frames per second, FPS) on ImageNet1k for different methods.

Models	Training	Inference
ResNet-50 [50]	**1204 FPS**	**1855 FPS**
ECA-Net [18]	785 FPS	1805 FPS
ResNet-50+CBAM [46]	472 FPS	1213 FPS
SENet-50 [40]	759 FPS	1620 FPS
SKNet-50 [17]	733 FPS	1578 FPS
ResNet-101 [50]	386 FPS	1174 FPS
ResNet-101+CBAM [39]	270 FPS	635 FPS
ResMLP-36 [35]	343 FPS	978 FPS
SENet-101 [40]	367 FPS	1044 FPS
SKNet-101 [17]	352 FPS	1002 FPS
Transformer local-attention (NesT-B) [43]	244 FPS	566 FPS
CDNet-50	794 FPS	1832 FPS
CDNet-101	372 FPS	1135 FPS

## Data Availability

Data are contained within the article.
